# Combined Surgical Management and Endovascular Repair of Aortic Arch Mycotic Pseudoaneurysm Secondary to Descending Necrotizing Mediastinitis

**DOI:** 10.1016/j.atssr.2025.05.013

**Published:** 2025-06-06

**Authors:** Ahmed J. AlAraibi, Ammar Shaaban, Muhammad Ashfaq, Husam Noor, Martin Maresch, Nazar Bukamal, Zaid Arekat, Habib Al-Tareif

**Affiliations:** 1 School of Medicine, Royal College of Surgeons of Ireland – Medical University of Bahrain, Busaiteen, Bahrain; 2 Department of Cardiothoracic Surgery, Mohammed bin Khalifa bin Salman Al-Khalifa Cardiac Center, Awali, Bahrain; 3 Department of Interventional Cardiology, Mohammed bin Khalifa bin Salman Al-Khalifa Cardiac Center, Awali, Bahrain; 4 Department of Vascular and Endovascular Surgery, Mohammed bin Khalifa bin Salman Al-Khalifa Cardiac Center, Awali, Bahrain; 5 Department of Cardiothoracic Anesthesia, Mohammed bin Khalifa bin Salman Al-Khalifa Cardiac Center, Awali, Bahrain

## Abstract

Descending necrotizing mediastinitis is a life-threatening infection, and in extremely rare instances it can erode into the aortic wall and lead to mycotic pseudoaneurysms. A 50-year-old man presented with chest pain, hoarseness, and dysphagia. Imaging revealed an aortic arch pseudoaneurysm and a mediastinal abscess containing multidrug-resistant *Salmonella.* Urgent surgical repair using deep hypothermic circulatory arrest allowed thorough debridement and patch closure. Two months later, suture line dehiscence was successfully managed by thoracic endovascular aortic repair. He recovered under prolonged antibiotic therapy. This case underscores the importance of early recognition and a multidisciplinary, staged approach to overcome both immediate and delayed complications.

Descending necrotizing mediastinitis (DNM) is a rare but highly aggressive thoracic infection that typically originates from oropharyngeal or deep cervical sources. It is characterized by rapid progression along fascial planes and often requires urgent surgical intervention. DNM can extend to involve major vascular structures, such as the aorta, thus placing patients at risk for aneurysm formation and possible rupture. Mycotic type among all forms of aortic aneurysm is estimated at 0.7% to 3%,^1^ and aortic arch involvement is extremely rare. Proposed mechanisms for development include septic embolization, hematogenous arterial wall seeding, and direct or lymphatic spread from nearby infected tissue.

Despite these established mechanisms, there is a paucity of literature describing mycotic pseudoaneurysm of the aorta specifically caused by DNM. The clinical course in our patient required both open surgical repair and delayed endovascular intervention, and this case highlights the complexity and urgency required in the management of these rare, life-threatening conditions.

A 50-year-old Bahraini man presented to the emergency department with a 1-week history of central chest pain, progressive hoarseness, and difficulty swallowing. He had a medical history of type 2 diabetes mellitus, hypertension, dyslipidemia, smoking, and a previous non–ST-segment elevation myocardial infarction treated with percutaneous coronary intervention to the obtuse marginal artery in April 2024.

On examination, the patient was hemodynamically stable. Auscultation revealed coarse inspiratory crackles and a pericardial rub. Because his electrocardiogram demonstrated hyperacute T waves in the anterior leads, he was transferred through the ST-segment elevation myocardial infarction hotline for urgent coronary evaluation. Angiography revealed a patent stent and no new obstructive lesions. An ear, nose, and throat examination revealed mild epiglottic edema and left vocal cord paralysis with incomplete glottic closure. Transthoracic echocardiography showed a moderate to large pericardial effusion with fibrinous layering and a reduced left ventricular ejection fraction (35%-45%). Pericardiocentesis drained 395 mL of serosanguinous fluid. A chest X-ray showed tracheal deviation to the right. Contrast-enhanced computed tomography of the chest demonstrated a 6 cm × 7 cm × 8.9 cm anterior mediastinal space-occupying lesion containing gas and fluid that was closely associated with an aortic arch pseudoaneurysm (Figure 1, Figure 2). Cultures of the pericardial fluid and blood both grew *Salmonella* spp resistant to several antibiotics, including amikacin, cefazolin, cefuroxime, and gentamicin.

**Figure 1:**
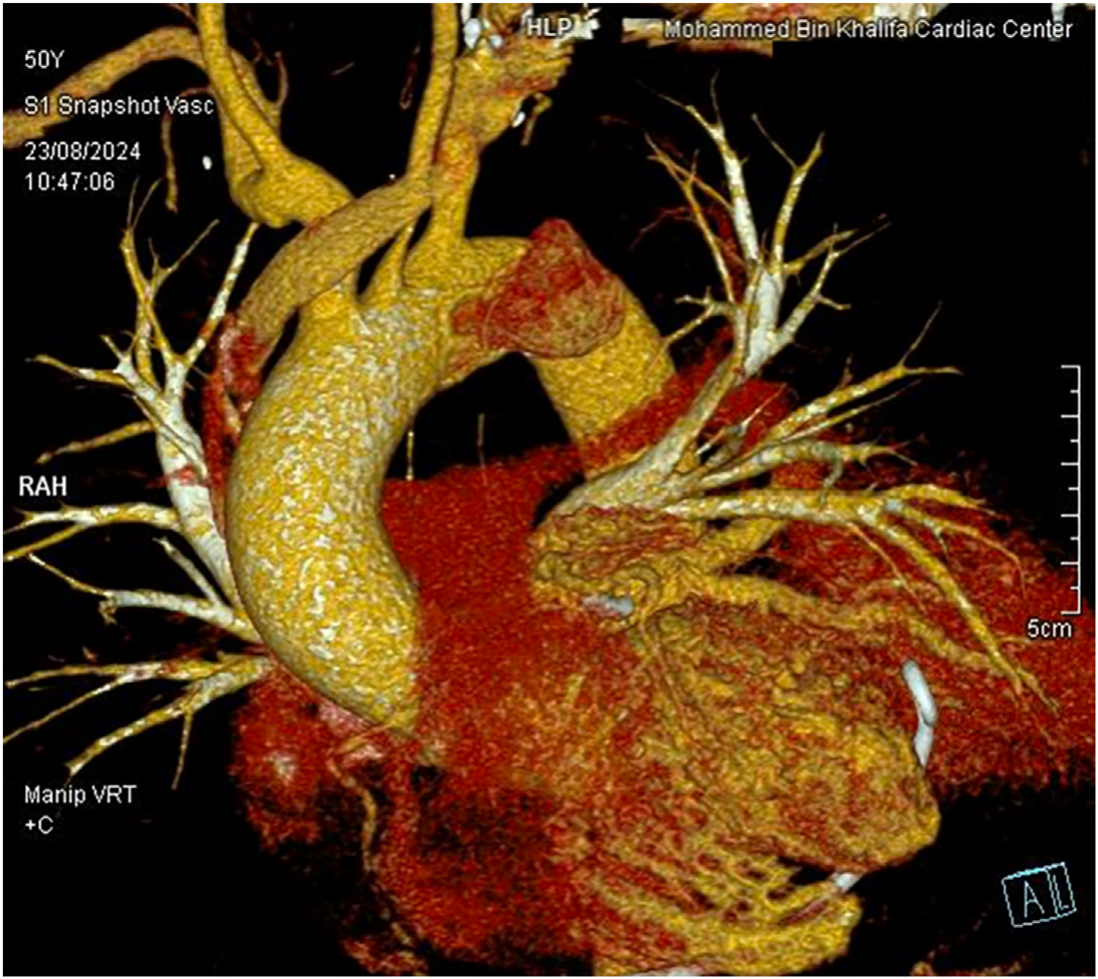
A 3-dimensional computed tomographic scan of the pseudoaneurysm. A 2.3 cm × 3 cm × 3.6 cm aortic arch pseudoaneurysm with a 13-mm was located on the anterior wall of the aortic arch at the same level as the origin of the left subclavian artery but not involving the arch vessels. (A, anterior; L, lateral; Manip VRT, manipulated volume rendering technique; RAH, right atrium of the heart; +C, contrast-enhanced.)

**Figure 2:**
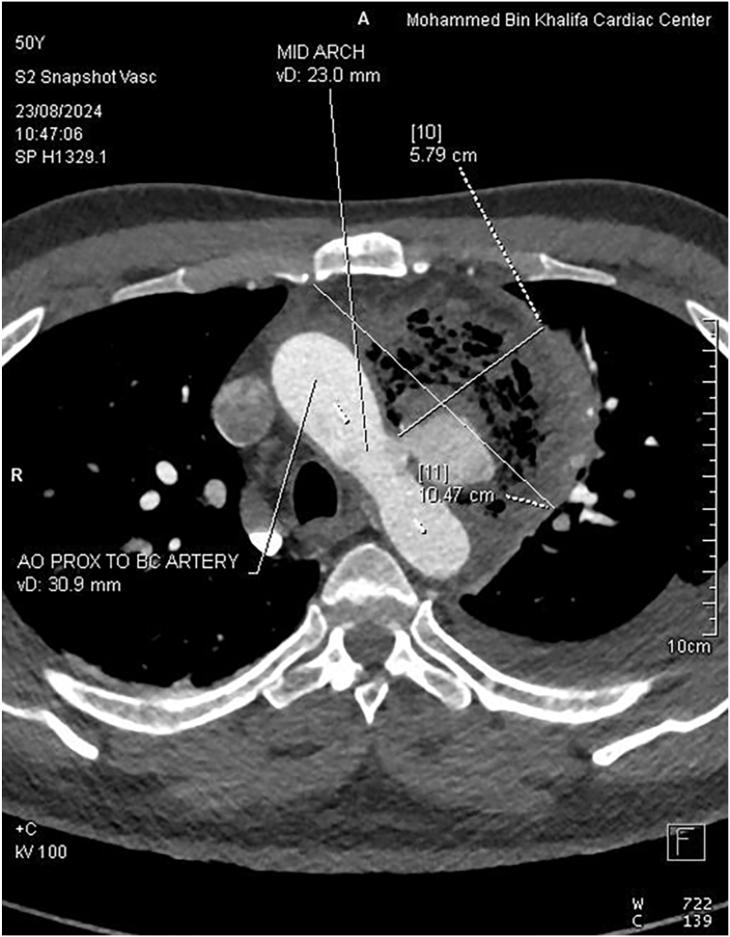
Contrast-enhanced computed tomographic findings. A well-defined lesion in the left anterior mediastinum measured approximately 6.0 cm × 7.0 cm × 8.9 cm and contained both gas and fluid. It was located adjacent to the aortic arch, which showed a pseudoaneurysm. (AO, aorta; BC, brachiocephalic; F, frontal view; PROX, proximate. R, right; +C, contrast-enhanced.)

In August 2024, urgent surgery was performed through median sternotomy and vertical pericardiotomy using bypass and deep hypothermic circulatory arrest. Antegrade cerebral perfusion was provided through the innominate artery. Intraoperative findings included friable mediastinal tissue, a 2.0-cm perforation in the distal aortic arch, and extensive abscess formation. Given these findings, a staged approach prioritizing infection control and vascular stabilization was chosen. Dissection of the mediastinal abscess and evacuation of the debris and fluid were performed, and tissue cultures were sent from the obtained tissue and aortic sac. These tissues grew the same organism as found in the blood cultures. The arch defect was addressed through a wide-margin resection, ensuring that only healthy aortic tissue was sutured to the patch, and then repaired with a double-patch technique. A polyester (Dacron, Invista) patch was placed internally and bovine pericardium was placed externally, secured with 4-0 pledgeted polypropylene (Prolene, Ethicon) sutures.

Postoperatively, the patient required continuous renal replacement therapy for acute kidney injury and intravenous anticonvulsant agents for a single seizure episode. He also experienced supraventricular tachycardia and atrial fibrillation, which were treated with intravenous amiodarone. He was extubated on postoperative day 6 and discharged home on day 19 with improved cardiac function (ejection fraction, 45%-50%) and minimal residual pericardial effusion. He was prescribed oral ciprofloxacin for an extended duration on the basis of culture sensitivities.

Two months postoperatively, the patient was readmitted with recurrent hemoptysis severe enough to warrant multiple blood transfusions. Imaging confirmed suture dehiscence at the distal aortic arch and stenosis of the proximal left subclavian artery. Given the high risk of reoperation in a previously infected and surgically altered field, thoracic endovascular aortic repair (TEVAR) was performed. Access was obtained through the right femoral and left radial arteries. A fenestrated stent graft was deployed across the aortic arch, with additional stenting of the left subclavian artery.

Hemoptysis resolved immediately after the procedure, and the patient remained hemodynamically stable and was started on 3 months of ciprofloxacin. At 6-month follow-up, the patient was clinically stable, with no recurrence of infection (Figure 3).

**Figure 3:**
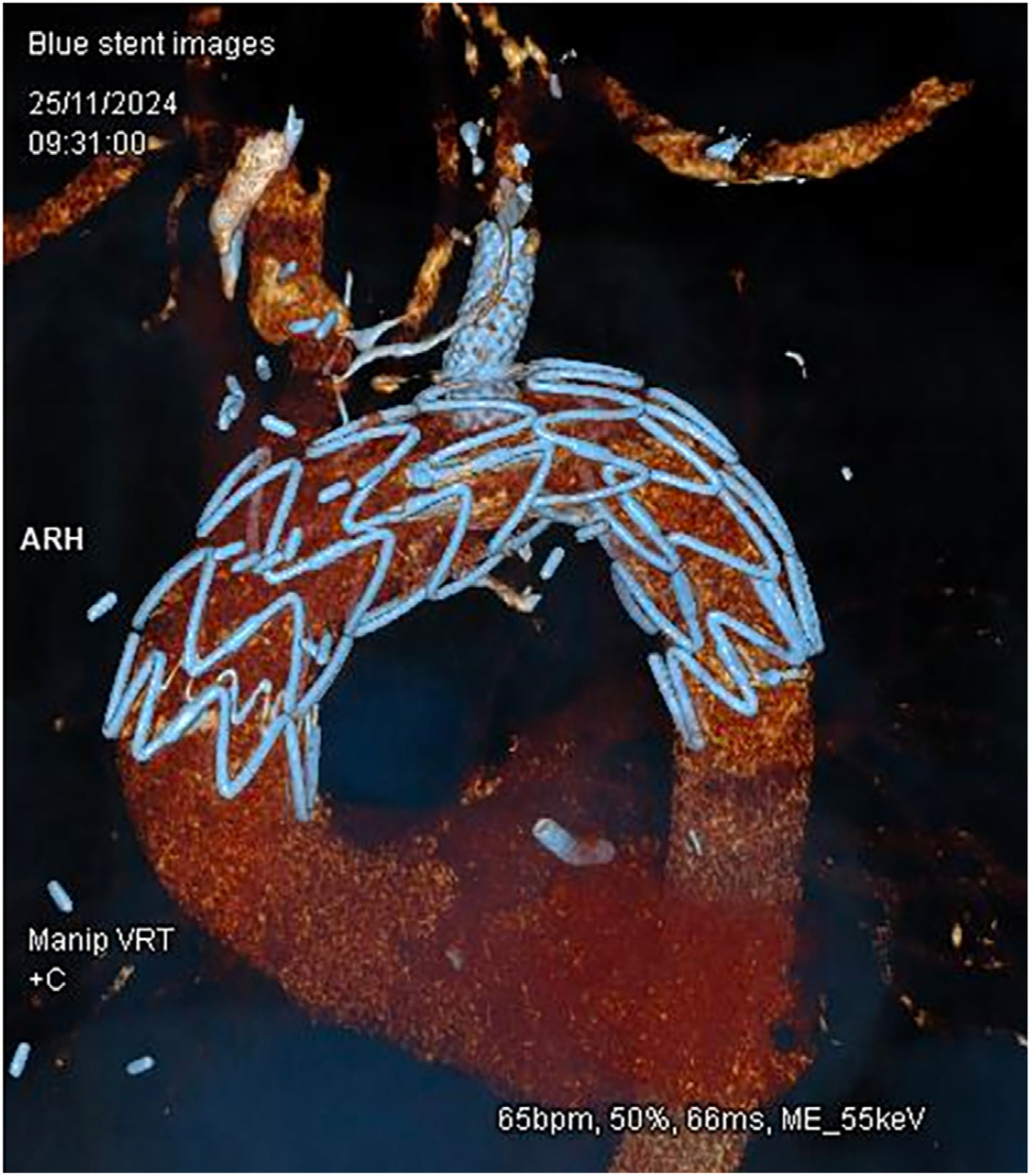
Postfenestrated thoracic endovascular aortic repair aortic arch. A follow-up 3-dimensional computed tomographic scan demonstrated that aortic graft was well deployed. Good flow to head and neck vessels with a patent stent to the left subclavian artery was noted. (ARH, aortic arch; bpm, beats/min; Manip VRT, manipulated volume rendering technique +C, contrast-enhanced.)

## Comment

Here we present the successful staged management of a case of DNM resulting in mycotic pseudoaneurysm, scenario rarely described in the literature. An initial open procedure was chosen to provide the advantage of debridement, which was the gold standard alongside an extended course of antibiotics to manage the mediastinal infection.^2^ However, when addressing the aortic arch defect, the debate centered on whether to proceed with patch repair or total arch replacement (TAR). Immediate TAR was deemed high risk given the potential for catastrophic complications, including early dehiscence related to the complexity of performing end-to-end anastomosis, uncontrollable hemorrhage, and graft infection. Existing literature has highlighted the increased risks associated with TAR in cases of infected aortic arch aneurysms, including high mortality, reinfection, and hypoxic-ischemic encephalopathy.^3^ Instead, we prioritized infection source control, followed by a conservative approach to stabilize the pseudoaneurysm using a patch repair technique.

Despite initial stabilization, the patient experienced suture line dehiscence, a recognized complication in up to 8% of aortic repairs.^4^ In our case, when the patient had a suture line dehiscence, the multidisciplinary team advised endovascular intervention to avoid the morbidity and mortality of redo sternotomy. TEVAR allowed immediate stabilization of the dehiscence, thereby preventing life-threatening rupture while avoiding the dangers of major reoperation in a previously infected field. Endovascular intervention was particularly suitable because the repair was entirely endoluminal and given that the patient showed no evidence of persisting infection.

Although *Salmonella* is a well-recognized pathogen in vascular infections, its role in DNM is exceedingly rare.^5^ In our patient, *Salmonella* was isolated from multiple sources, thus confirming its systemic dissemination and direct role in both the mediastinal infection and the pathogenesis of the mycotic pseudoaneurysm. The rarity of *Salmonella* as a cause of DNM combined with its more recognized association with vascular infections highlights the unique interplay between the organism’s virulence and the compromised immunity of the host in this case, an interplay that was crucial in driving clinical management with the appropriate antibiotic regimen.

In conclusion, aortic arch pseudoaneurysm secondary to DNM is an extremely rare but severe entity that may require both open surgical and endovascular strategies. In this case, emergency surgery stabilized the pseudoaneurysm and cleared the mediastinal infection, whereas delayed complications were addressed using TEVAR when reoperation was deemed excessively hazardous. Long-term antibiotic therapy, regular imaging, and close hemodynamic surveillance were critical to ensuring a favorable outcome. This case underscores the importance of a multidisciplinary approach and the need for sustained vigilance in managing complex aortic infections.
